# The Present and Future of Virology in the Czech Republic—A New Phoenix Made of Ashes?

**DOI:** 10.3390/v14061303

**Published:** 2022-06-14

**Authors:** Tomas Ruml

**Affiliations:** Department of Biochemistry and Microbiology, University of Chemistry and Technology, 166 28 Prague, Czech Republic; tomas.ruml@vscht.cz; Tel.: +420-220-44-3022

The Czech Republic, a part of the former Czechoslovakia, has been at the forefront of several research directions in virology, genetics and physiology. For the latter disciplines, the names of Johan Gregor Mendel (1822–1884) and Jan Evangelista Purkynje (1787–1869), the two Austro-Hungarian scientists born in the territory of the contemporary Czech Republic, can be named. 

Modern times are proud of important Czech virologists, such as Karel Raška (1909–1987) who was a Director of the WHO’s Division of Communicable Disease Control since 1963. His new concept of eliminating smallpox was adopted by the WHO in 1967 and eventually led to the eradication of smallpox in 1977. Raška was also a strong promoter of the concept of epidemiological surveillance of diseases, which was adopted in 1968 and has since become a standard practice in epidemiology. In 1984, he received the Edward Jenner Medal awarded by the Royal Society of Medicine. Another brilliant scientist who must be named is Jan Svoboda (1934–2017), a pioneer of retrovirology, brilliant scientist, and, for many years, a living legend in the broader field of retroviruses, tumor viruses, and oncogenes. He created the hypothesis that retroviruses can transform healthy mammalian cells into cancerous ones. This had a direct impact on the discovery of reverse transcription described by Howard Temin, who won the Nobel Prize for this discovery. Last but not least, Antonín Holý (1936–2012) was a pioneering Czech organic chemist who specialized in the development of important antiretroviral drugs, especially nucleotide analogues used in the treatment of HIV-1 and hepatitis B. He was involved in the creation of numerous very effective and commercially successful drugs for the treatment of AIDS and other viral diseases.

The current coronavirus pandemic has shown the importance of virological research. For historical reasons, the Czech Republic has no scientific institution where such research is concentrated. The former Institute of Virology of the Czechoslovak Academy of Sciences was established on 1 January 1953 in Bratislava (current Slovakia). The institute studied an important concept of the natural focality of diseases and the ecological problems of viruses and viral diseases. The Institute also participated in the discovery of tick-borne encephalitis virus transmission via goat milk and dealt with its structure, antigenic characteristics, and immunology. However, after the Dissolution of Czechoslovakia in 1 January 1993, resulting in split of the federal republic of Czechoslovakia into two independent countries, the Czech Republic and Slovakia, the Institute remained on its premises in the capital of Slovakia: Bratislava. This left the Czech Republic without any coordinated virological research. Only recently, due to the rising virological threats, has the discussion been initiated on establishing a virological institute in the Czech Republic. It became apparent that the understanding of viral properties and modes of spreading and treatment may not only help to bring significant discoveries leading to development of new antiviral strategies and compounds, but it may also facilitate communication between the scientific community and the institutions responsible for setting epidemiological rules. The concept of the currently emerging institute is based on the financial support of selected excellent virological groups established at several Universities and Institutes of the Academy of Sciences in the Czech Republic. Thus, the Institute of Virology will consist of 25 groups established at eight research institutions and coordinated by an executive director (Zdeněk Hostomský) and scientific director (Pavel Plevka). The concept of independent but mutually coordinated research groups at several institutions will profit from complementary disciplines and access to unique state-of-the-art facilities available at individual institutions. The integral part of the project is the sharing of know-how, intense student and staff communication and seminars. It should be mentioned that a minor part of the Institute is devoted to drug-resistant bacteria, which have recently become a similarly important issue as viruses. Moreover, both virology and bacteriology share many common methodological approaches. It is expected that the combination of these two scientific disciplines under the umbrella of one institution will be mutually inspiring.

The research will be grouped into three major areas: (a) interactions between pathogens and hosts, (b) immunity against viruses and bacteria and (c) the development of drugs against viral and bacterial diseases The major focus areas of selected Czech virological laboratories, whether or not they are included in the Institute, are shown below (listed alphabetically according to the last names of the heads of the groups).

The Laboratory of Structural Biology at the Institute of Organic Chemistry and Biochemistry (head Evžen Bouřa) is focused on proteins essential for viral replication, including both host factors and viral enzymes [1]. For instance, some lipid kinases and/or lipid transport proteins are hijacked by viral proteins. Subsequently, they assemble into large multiprotein complexes that modify the membrane; this changes the membrane’s chemical composition, which leads to altered biophysical properties. They mainly use protein crystallography and other biophysical methods to understand the structure and function of these membrane-modifying enzymes in detail. They have focused on phosphatidylinositol 4-kinase B (PI4KB), which is an essential host factor for a variety of +RNA viruses, such as HCV, poliovirus and Coxsackie virus. The lipid it produces, phosphatidylinositol 4-phosphate (PI4P), is a hallmark of viral replication organelles (ROs) of these viruses. The crystal structures of these enzymes with small-molecule inhibitors were solved in collaboration with the Nencka group, and the structural information was used to develop PI4KB inhibitors that exert nanomolar inhibition activity and have the potential to be used as virostatics [2]. Another topic studied is the molecular mechanism of PI4KB membrane recruitment by the Golgi-resident protein ACBD3 and other viral proteins, especially the methyltransferases (MTases) and polymerases (RdRp) of SARS-CoV-2 and various flaviviruses [3]. A series of crystal structures of coronaviral and flaviviral polymerases was solved [4,5] in a complex with inhibitors that were prepared by the Nencka group. They also biophysically characterized the RdRps from SARS-CoV-2 and various flaviviruses [4,5].

The goal of the Klara Grantz Saskova group at the same Institute is to study the mechanisms of HBV escape from intrinsic and innate immunity for the discovery of new anti-viral drugs. To this end, they study (i) the effects of microenvironments of HBV-infected hepatocytes, including that of extracellular vesicles, on PBMCs; (ii) the interplay between promyelocytic leukemia protein nuclear bodies and HBV in the nucleus; and (iii) interactions of the HBV core (HBc) protein with the proteins of the host to inhibit virus replication. In vitro high-throughput screening assays are used to identify small compounds affecting the respective protein–protein interactions.

Research in the Laboratory of Viral and Cellular Genetics at the Institute of Molecular Genetics in Prague (head Jiří Hejnar) is focused on retroviruses, the interactions of viruses with the host cell and the control of retrovirus replication by the host cell’s factors, whose presence or absence in the cell defines the cell’s permissiveness or resistance to retroviral infection. An example of such virus dependence factors are entry receptors in a model of avian leukosis virus. This virus diversified into several subgroups using different cell surface receptors [6] and is thus suitable for the research of virus–host co-evolution and for comparative studies. It led to the discovery of numerous virus-resistant receptor alleles and the construction of the chicken lines resistant to ALV-J [7], an important pathogen of domestic chicken. On the other hand, host cells produce factors which restrict a virus at various steps of its replication cycle. These are defined for HIV-1 in human cells but not for other retroviruses. Again, ALVs represent an opportunity to identify the new restriction factors and study them in terms of virus–host co-evolution [8]. 

In the field of antiviral innate immunity, ALVs as a model virus and its natural host, chicken, aided in the discovery of “hidden” genes in microchromosomal regions extremely enriched in GC, including the restriction factor tetherin and the innate immunity factor TNFalpha [9]. The analysis of RIG-like receptors in birds presented here is in line with this branch of our research. The focus also includes endogenous retroviruses, their identification by genomic tools and the exaptation of virus genes for cellular functions. Retroviral vectors have been developed for studies on the epigenomics of retrovirus integration and somatic hypermutation [10]. The results extend into the applied research of gene therapy, the latency of HIV-1 and gene editing in chickens. 

The Medical Zoology Laboratory of the Institute of Vertebrate Biology, Czech Academy of Sciences (head Zdeněk Hubálek), was originally founded by Bohumír Rosický as the Laboratory for research on natural focality of viral and bacterial diseases at the Parasitology Institute Prague, Czechoslovak Academy of Sciences, in 1972. The laboratory is active in the field of arboviruses. For instance, Ťahyna orthobunyavirus was isolated from mosquito larvae and from the blood of sick children in south Moravia by the first lab head Vojtech Bárdoš in 1975 [11], and tick-borne encephalitis virus was isolated from Ixodes ricinus ticks in the holiday resort at Vranov (1978) and from a bank vole (Myodes glareolus) in Austrian Styria in 1977 [11]. Bhanja virus we recovered in southeast Bulgaria from Haemaphysalis punctata ticks and then in eastern Slovakia in sheep and goats, and later (1986–1988) even isolated it there from Dermacentor marginatus ticks [12]. After the big 1977 flood, South-Moravian mosquitoes surprisingly yielded, in addition to Ťahyňa virus, also the West Nile flavivirus (WNV, a new lineage 3-Rabensburg). The spread of WNV has continued, causing disease in few humans but also death in a number of raptors (mainly goshawks) in the Czech territory [13,14,15]. Also notable is the discovery of another pathogenic Usutu flavivirus in South-Moravian mosquitoes and dead blackbirds, namely, Turdus merula.

The main focus of the Jan Konvalinka group at the Institute of Organic Chemistry and Biochemistry, Academy of Science of the Czech Republic, is the identification, validation and characterization of traditional or novel therapeutic targets for the diagnosis and treatment of viral diseases and various tumors. They combine several approaches to achieve these goals, including synthetic chemistry, chemical biology, structural biology and biochemistry. The models studied involve HIV, influenza, Zika, Dengue and other human viruses, as well as prostate cancer and glioblastoma. The methods used span from organic synthesis to molecular modelling and medicinal chemistry, recombinant protein production in various organisms, enzymology and X-ray and NMR structure analysis to mammalian cell cultures and xenografts of human tumors and transgenic mice. Recently, the Konvalinka group developed novel chemical biology tools based on the conjugates of specific ligands with either biocompatible polymers or DNA oligonucleotides (iBodies, DIANA). They could be used for the identification, isolation, visualization and quantification of a variety of protein targets and for high-throughput testing of their inhibitors and ligands. Using these tools, they search for novel inhibitors of known target enzymes and try to identify new therapeutic targets.

The Laboratory of Virology at the Institute of Experimental Botany (head Tomáš Moravec) focuses on the study of plant viruses and the management of viral diseases in plants. In addition to the development of tools for reliable virus detection, the main focus is on the area of molecular farming using viruses and their parts to express vaccines, enzymes and antibodies in plants. The programmable CRISPR/Cas9 nucleases in combination with viruses is used to develop gene-editing strategies for marginal crops. Over the years, many plant viruses, and their infectious clones, namely, Tobacco mosaic virus strains U1 and Cg8, Potato virus X, Tobacco rattle virus, Apple latent spherical virus and Bean Yellow Mosaic Virus, were modified for the GoldenBraid convention. This greatly simplifies the exchange of parts and genes between viral vectors. A newly established joint laboratory with the Institute of crop protection will help to conduct both basic and applied research in the field of plant virology.

The Laboratory of Structural Virology, headed by Pavel Plevka at CEITEC, Masaryk University, focuses on the cryo-electron microscopy of virus particles and the cryo-electron tomography of virus-infected cells. They aim to structurally characterize the replication cycle of the viruses and their interactions with components of the immune system. They determined the structures of several enteroviruses and described their genome release mechanism, which involves capsid opening [16,17]. Furthermore, they also study honeybee viruses from the families Iflaviridae and Dicistroviridae [18]. The group of Pavel Plevka determined the structure of the tick-borne encephalitis virus and its mechanism of neutralization by monoclonal antibodies [19]. Other areas of research interest include tailed bacteriophages that infect pathogenic bacteria and, therefore, could be used in phage therapy [20]. They study the dynamics of phage infection of biofilms and the replication of phages in infected cells.

The research of Iva Pichová laboratory of Viral proteins at the Institute of Organic Chemistry and Biochemistry in Prague has predominantly focused on the functional and structural characterization of proteins regulating the key steps in the lifecycle of different viruses, the identification of cellular proteins involved in virus replication and persistence development. The group investigated the maturation and assembly of retroviruses such as Mason-Pfizer monkey virus, HIV-1 and Mouse mammary tumor virus. Currently, the laboratory focuses on regulations of molecular mechanisms of hepatitis B virus proteins replication, cccDNA transcription activation and maturation of precore protein HBe during the HBV life cycle, with the goal to identify novel targets for drug development.

The virology group at the University of Chemistry and Technology, Prague (head Michaela Rumlová), is focused on several aspects of the replication cycle of selected members of viruses from the families Retroviridae, Flaviviridae, and Coronaviridae. The research is focused on the process of assembly, during which viral proteins and viral genomic RNA form both mature and immature viral particles, as well as on the structure of viral proteins, the functional significance of their structural domains, their mutual interactions, interactions with RNA and membranes and the transport of viral proteins or whole intracytoplasmic viral particles in an infected cell [21]. Attention is also paid to the characterization of the interactions among viral and host cell proteins (and small molecules) needed by the virus to accomplish its replication cycle. To screen potential inhibitors, a variety of in vitro high-throughput assays for testing molecules interfering with the assembly process viral enzymatic activities, such as RNA-dependent RNA polymerase, RNA helicase and reverse transcriptase, have been established [22,23,24,25].

The Laboratory of Arbovirology (head Daniel Růžek) is a joint research unit of the Institute of Parasitology, the Biology Centre of the Czech Academy of Sciences in Ceske Budejovice, and the Veterinary Research Institute in Brno, Czech Republic. The laboratory studies the molecular basis of diseases caused by tick-borne encephalitis virus and other flaviviruses, with particular emphasis on the interface between pathogenesis and host immunity and on the development and testing of new antiviral agents and vaccines. The laboratory contributed significantly to the characterization of tick-borne encephalitis virus structure [19] and the discovery of novel human monoclonal antibodies to tick-borne encephalitis virus with prophylactic and therapeutic potential [26]. Several key aspects of the pathogenesis of tick-borne encephalitis, including the immune response to infection, have been described by the laboratory, including changes in the blood–brain barrier permeability during infection, interaction of the virus with cells that form the blood–brain barrier and with primary human neurons and astrocytes, characterization of immunopathological features during disease, etc. The laboratory also pioneered research on antiviral agents effective against tick-borne encephalitis virus [27] and other flaviviruses and developed the first veterinary tick-borne encephalitis vaccine candidate [28]. More recently, the laboratory has begun research into the biology and pathogenesis of SARS-CoV-2 and has been involved in the development of a new mouse model for COVID-19 and in the discovery of the first bispecific antibodies that neutralize SARS-CoV-2 [29].

The Laboratory of Immunotherapy at the Faculty of Science, Charles University (head Michal Šmahel), combines the activation of innate and adaptive immunity to treat tumors induced by human papillomaviruses. To enhance the efficacy of cancer immunotherapy, mouse tumor models are developed and used for the examination of immunotherapy [30,31,32]. These models are characterized by immune escape mechanisms that hamper an antitumor effect of immunotherapy in clinical trials; particularly, the downregulation of MHC class I molecules is analyzed as one of the most frequent mechanisms of tumor escape from host immunity. To study factors that influence the effect of cancer immunotherapy, immune cells that infiltrate tumors with various MHC class I expressions are characterized, and immune reactions are analyzed [33,34]. 

The Laboratory of Molecular and Tumor Virology at the Faculty of Science, Charles University, headed by Ruth Tachezy, is involved in the studies of small non-enveloped DNA viruses associated with tumors in humans. Apart from human papillomaviruses, the laboratory team focuses on human polyomaviruses and other small DNA viruses (anelloviruses or human bocavirus). The team focuses on molecular epidemiology of tumor viruses and developed several approaches to characterize the viral etiology of tumors [35,36,37]. Recently, the group is investigating tumor microenvironments with the aim to explain the prognostic advantage of patients with virally induced tumors and evaluate immune prognostic markers as well as therapeutic markers of small inhibitory molecules [38]. Furthermore, the group also develops tools for the analyses of viromes of both animals and humans and studies the interplay between the virome composition and infection of other pathogens on health and disease [39]. 

Jan Weber’s laboratory is focused on interactions of the hepatitis B virus core protein with host cell proteins. In particular, they are interested in the characterization of proteins and cellular pathways involved in (i) the epigenetic regulation of transcription, (ii) ubiquitin–proteasome degradation and (iii) post-translational modifications [40,41,42]. Furthermore, attention is paid to the effect of the microenvironment of HBV-infected hepatocytes on the function of plasmacytoid dendritic cells [43]. In addition, they explore strategies to target the virus’s attachment and entry to the cells. Many viruses use heparan sulfate proteoglycan for initial attachment onto the cell surface. Using various gold and silver nanoparticles with multi-sulfonated ligands, we characterize this attachment process to find new ways to block the virus’s entry in the cells [44]. Next, we are interested in the role of specific aGPCR and cellular proteins in aGPCRs pathways involved in viral infections of mammalian cells. Finally, we perform screening of antiviral compounds against a variety of DNA and RNA viruses.

The Laboratory of Trypanosomatid Biology at the Life Science Research Centre, University of Ostrava (head Vyacheslav Yurchenko) is focused on investigating trypanosomatids sensu lato. These unicellular flagellates are obligatory parasites, causing a number of human, domestic animal and plant diseases [45]. Because of their diversity, adaptability to dramatically different environmental conditions and omnipresence, these protists have major impact on all biotic communities. One of the research directions in the laboratory concerns studies of symbiotic associations between trypanosomatids and viruses [46]. The best-studied cases are of Leishmania RNA viruses (Leishmaniavirus spp., LRVs of the family Totiviridae). Their presence is linked to the increased metastatic potential, parasite burden, immune response in mouse models of leishmaniasis and frequent treatment failures [47]. Other groups of RNA viruses infecting trypanosomatids are bunyaviruses and narnaviruses, along with some viruses of more restricted distribution [48]. The applied methodological approaches include whole-genome and whole-transcriptome sequencing followed by the phylogenomic analyses of viruses and their respective trypanosomatid hosts [49]. 

Public Health Institute, Ostrava (PHIO), is a governmental institution providing services in the field of health promotion and protection. It comprises several National Reference Laboratories (NRL), including the NRL for arboviruses (head Hana Zelená). The NRL for arboviruses works as a part of the Department of Virology, providing laboratory diagnostics of a wide spectrum of human viral diseases, including infections with arboviruses and rodent-borne viruses (e.g., tick-borne encephalitis virus, West Nile, Zika, dengue, yellow fever, Japanese encephalitis, chikungunya, Tahyna, Sindbis, phleboviruses, hantaviruses), developing new in-house diagnostic tools and confirmatory assays. Their main research interests are in the diagnostics of arboviruses and rodent-borne viruses, imported and emergent viral diseases, viral infection of the nervous system and electron microscopy. Other professional activities are consulting activities in the field of clinical microbiology and antibiotic therapy. 

I sincerely thank the authors and reviewers for their efforts when contributing to the collection of the articles in this Special Ossue. I believe that the Special Issue has provided a fresh insight into current trends in virology in the Czech Republic and can be an impulse for establishing new collaborations.
